# Connections between Children's Eating Habits, Mental Health, and Parental Stress

**DOI:** 10.1155/2022/6728502

**Published:** 2022-04-12

**Authors:** Marco Tommasi, Francesca Toro, Alessandra Salvia, Aristide Saggino

**Affiliations:** Department of Medicine and Aging Sciences, University of Chieti Pescara, Chieti, Italy

## Abstract

**Background:**

Obesity and eating disorders are increasing in occidental countries and can undermine physical and psychological health. Therefore, preventing the insurgency of unhealthy eating habits in childhood is fundamental. Parents can play an important role in assisting pediatricians, psychiatrists, and clinical psychologists in the diagnosis of eating disorders because they have an active role in observing and assessing the quality of their children's eating habits.

**Methods:**

In our study, we collected data from a sample of children (*n* = 125) and their parents (*n* = 161) without symptoms related to eating disorders. Parents assessed the eating habits, behavior problems, and mental health of their children and parental stress. In addition, we measured body mass index, anxiety, and lifestyle in children. Data were analyzed with bivariate correlation and MIMIC models.

**Results:**

Both mothers' and fathers' assessments of children's eating habits are reliable. Unhealthy eating habits are connected with children's behavioral problems and parental stress. We did not find significant differences in feeding styles and ways of assessing the quality of eating habits between mothers and fathers. Our study showed greater sensitivity of mothers toward the physical fitness of their children rather than fathers.

**Conclusions:**

Mothers and fathers both proved to be good observers of their children's eating behavior, and they could cooperate with medical and psychological operators in preventing the risk of obesity.

## 1. Introduction

According to the National Health and Nutrition Examination Survey [1], during the period 2017–2018, the prevalence of severe obesity among USA adults was 9.2%. Women had a higher prevalence of severe obesity (11.5%) than men (6.9%). The prevalence was highest among adults aged 40–59 (11.5%), followed by adults aged 20–39 (9.1%) and adults aged 60 and over (5.8%). Moreover, in Europe, the problem of obesity is evident. To date, almost 60% of the population of the OECD countries is overweight, and 25% have severe obesity [2]. Average rates of adult obesity in OECD countries have increased from 21% in 2010 to 24% in 2016, so 50 million people are now obese [2]. In 2017, in Italy, obesity affects a total of 10.5% of the population over the age of 18, 14.5% among those over 65. This condition is found more frequently among residents of the South (11.8%), reaching the highest share in Sicily (12.6%); [3].

Correct eating habits prevent people from developing eating disorders, which, in turn, can undermine physical health. In fact, obesity is associated with a high risk of heart attack, arterial hypertension, hyperglycemia, high cholesterol level, and diabetic hyperinsulinemia [4]. In children, obesity can lead to chronic inflammation, hypothyroidism, and hypogonadism [4]. Obesity and eating disorders, however, affect also mental health and psychological well-being because a bad diet is associated with a low quality of life and a low level of mental health [5–9].

Therefore, it is important that people develop good eating habits, especially during their childhood and adolescence. Many studies showed the important impact that parents have on children's eating habits. In particular, parents' well-being and mental health resulted to have an impact on children's eating behavior [10, 11]. Some studies showed that if children are left alone in selecting their food, they tended to select food with a high level of sugar [12]. Without the intervention of parents, particularly mothers, children tend to choose food more on the basis of its hedonistic characteristics than on the basis of its healthful effect on the body [12, 13]. The parental contribution to helping children find a healthy diet is indisputable [14–16]. Parents have the responsibility for providing children with an array of healthful food, while children have the responsibility for how much she/he eats [17].

Recent research showed that public programs for interventions against childhood obesity through schools cannot be efficient without considering other sources of support for a healthy lifestyle, particularly family [18]. Parents play an important role in supporting and helping their children to keep healthy dietary habits. Some studies showed that low parental competencies and lack of control in children's nutrition have negative effects on children's dietary habits [19]. Other studies showed that parents' underestimation of children's obesity, parents' pressure to eat, and low parent's level of monitoring have negative effects on the selection of healthy food for children [20]. Parents' attitude toward children's dietary habits has an important effect on children's nutrition quality [21].

Even if research showed that parents can play an important role in preventing the appearance of unhealthy eating habits in their children and in supporting healthy eating habits [15], it remains necessary to know their ability in assessing the quality of children's eating habits, whether these habits have some relations with children's mental health, and whether these habits can generate stress for parents.

Because other studies showed that children's health conditions can affect parents' attitude toward their eating habits [20, 21], we also took into consideration the psychological state of the children, particularly anxiety, and their physical conditions, particularly the body mass index (BMI), to see if there is a relationship between children's physical and psychological characteristics and the assessment of their eating habits by parents. Children with unhealthy eating habits should have an abnormal BMI (BMI >30 for obesity and <18 for underweight) and a lower level of psychological well-being indicated by excessive anxiety [10].

We have formulated different hypotheses. Our first hypothesis (H1) is that children's eating habits are affected by their physical and psychological conditions. In particular, this hypothesis is divided into two subhypotheses:

  H1a: Children's eating habits are affected by children's BMI (the higher the BMI, the more unhealthy are children's eating habits).
  H1b: Children's eating habits are affected by their anxiety (the higher the anxiety, the more unhealthy are children's eating habits).

Our secondary hypothesis (H2) is that children's unhealthy eating habits are connected to children's mental health because children with unhealthy habits should show psychological or behavioral disorders more intensely [5–7] and connected to parental emotive reactions because children with unhealthy habits tend to increase the level of parental stress [10]. This hypothesis is divided into two subhypotheses:

  H2a: Children's mental health is negatively affected by their unhealthy eating habits.
  H2b: Parental stress is increased by children's unhealthy eating habits.

In addition, considering the fact that there are some studies showing differences between attitudes and reactions of mothers and fathers toward children's eating habits [14, 16, 22], we decided to analyze whether there are some differences between mothers' and fathers' assessment of their children's behavior in relation to eating habits.

## 2. Methods

### 2.1. Participants

For our study, we selected a sample of parents and their children from a population attending a local scholastic complex in the central part of Italy (Abruzzo) dedicated to primary education. The scholastic complex consisted of two school buildings, both of which consisted of primary classes. We have directed our attention to primary school children because not only this period is fundamental for the development of healthy eating habits [15, 17, 23] but also because children of this age can answer structured questionnaires. A total of 125 children (50.5% females) and 161 parents (57.1% females) participated in the research. Children's age ranged from 8 to 11 years (mean age = 9.50; SD = 0.604); parents' age ranged from 28 to 57 years (mean age = 43.10; SD = 5.322). The majority of parents were employed as dependent or autonomous workers (45%) or as employees in administrative or commercial jobs (21%) and had a high school degree (51%—see Table 1 for these and other demographic data).

### 2.2. Materials for Child Assessment

The materials used for the surveys on children consisted of tools for anthropometric and psychological assessment. The anthropometric assessment was done with a meter for measuring body stature and a scale for measuring body weight. These measures were then used to assess the BMI of children in relation to their age. Psychological assessment was done by asking children to compile a short questionnaire, in which we asked them if they played sports, if they liked eating, if they were judged fat by their classmates, and if they were satisfied with the quality of their relationship with their parents by asking how much time the mother and father spent with them. Finally, in the questionnaire, we included two scales taken from the Screen for Child Anxiety Related Emotional Disorders (SCARED) [24]. These subscales, each comprising five items, measured the children's general anxiety level (Gen Anx) and separation anxiety (Sep Anx). The scores on the anxiety scales were then converted into values from 0 (no anxiety) and 2 (a lot of anxiety). Moreover, a global score of anxiety (Tot. Anx) was estimated for children.

### 2.3. Materials for Parent Assessment

In the questionnaire for parents, in addition to gender, age, and other personal data (educational qualification, level of income, type of profession, and marital status), some questions were asked about their lifestyle and diet and the way in which they fed their children. Regarding lifestyle, parents were asked if they played sports, if they followed a diet, and if they were overweight. Regarding the way in which they feed their children, they were asked if they encouraged their children to finish the meal, if they tended to cook the dishes preferred by their children, and if they let their children decide the food for lunch or dinner. After these questions, parents had to fill in three standardized questionnaires to assess the eating habits of their children, their level of stress, and the presence of behavioral and psychological disorders in their children.

### 2.4. The Children's Eating Behavior Questionnaire (CEBQ) [25]

The CEBQ is a parent-report questionnaire designed to assess eating styles related to obesity risk. The questionnaire is composed of 35 items divided into 8 subscales, each of which assesses a particular eating style: food responsiveness (FR), emotional overeating (EOE), enjoyment of food (EF), desire to drink (DD), satiety responsiveness (SR), slowness in eating (SE), emotional undereating (EUE), and food fussiness (FF). FR evaluates if the child has a positive reaction to food (e.g., “My child's always asking for food”); EF evaluates how much the child likes to eat (e.g., “My child enjoys eating”); EOE evaluates the child's tendency to overeat when agitated or worried (e.g., “My child eats more when anxious”); DD evaluates the child's tendency to drink (e.g., “My child is always asking for a drink”); SR evaluates the level of satiety reached by the child during meals (e.g., “My child gets full up easily”); SE evaluates how fast the child is in finishing eating (e.g. “My child eats slowly”); EUE evaluates the child's tendency to eat when he is agitated, sad, or angry (e.g., “My child eats less when s/he is angry”); and FF evaluates the child's tendency to be picky when eating (e.g., “My child is difficult to please with meals”). Responses are on a Likert scale from 0 (never true) to 2 (certainly true).

### 2.5. The Parenting Stress Index-Short Form (PSI-SF) [26]

The PSI-SF is composed of 36 items, with Likert scales ranging from 1 (strongly disagree) to 5 (strongly agree). The test is divided into 3 subscales, each made up of 12 items, which measure different aspects of parental stress: parental stress (PD), parent-child dysfunctional interaction (PCDI), and the difficult child (DC) subscale. The PD subscale measures the parent's overall stress level; the PCDI subscale measures the quality of the parent-child interaction, in the sense that the higher the score, the lower the quality of the interaction; and the DC subscale measures the child's level of problematic behavior. There is also a global score on the PSI-SF scale.

### 2.6. The Child Behavior Check List-6/18 (CBCL 6/18) Parent Form [27]

The CBCL 6/18 is a questionnaire composed of 113 items that explore various areas: activity, interest, attention, fear, play, interaction with peers and adults, anxiety, somatic conditions and problems, mood, aggression, and affective responsiveness and response to changes. CBCL is divided into 9 subscales that are: introversion (INT), psychosomatic disorders (SOM), anxiety and depression (ANX), socialization (SOC), ideation (IDE), attention (ATT), deviant behavior (DEV), aggression and hyperactivity (AGG), and other problems (OTHER). Scores are on a Likert scale from 0 (not true) to 2 (completely true). The sum of the INT, SOM, and ANX subscales generates the “internalizing” CBCL score (INT-CBCL), while the sum of the DEV and AGG subscales generates the “externalizing” CBCL score (EXT-CBCL). The sum of INT-CBCL, EXT-CBCL, and OTHER subscales gives the global CBCL score. For each subscale of the CBCL and for the internalizing and externalizing scales, there are cutoff values to determine if the child is normal, borderline, or clinical [28].

### 2.7. The Marlowe–Crowne Scale (MC) [29]

The MC is a control scale for assessing social desirability. If there is a positive correlation between the MC and other test scores, then subjects tend to overestimate their trait or characteristics; if there is a negative correlation, then subjects tend to underestimate their traits or characteristics. We used a short form of the MC [30], with nine items on a Likert scale from 1 (completely false) to 5 (completely true).

### 2.8. Procedure

Children and parents were recruited through a series of meetings between the research staff and families organized in collaboration with the teachers of the scholastic complex. In these meetings, the purposes and procedures of the research were explained both to parents and teachers of the 4th and 5th grade classrooms. In these meetings, we asked parents who wanted to participate to communicate their availability to the teachers who had the task to write on a list the names of the parents who accepted to participate. Participation was voluntary and not paid. After having collected the subscriptions from the research volunteers, some members of the research staff (A.S. and F.T.) went regularly to the school on specific days of the week, according to teachers' consensus and availability, to measure the BMI of the children whose parents accepted to collaborate and to administer them a short questionnaire. When children ended the compilation, they were given another questionnaire to be filled out by their parents at home. Parents were also given instructions for returning the questionnaire within a certain period of time (two weeks). Parents' compiled questionnaires were returned by the children to their teachers. Subsequently, the researchers moved on to the classes involved in the study to collect all the questionnaires. The total time required for administering and collecting all the questionnaires was approximately 3–4 months. The data collection occurred in the last months of 2019, shortly before the lockdown imposed by the COVID-19 pandemic. Parents signed informed consent for their participation. In the consent sheet, they were given information about respect for privacy, according to the Italian and European laws (Italian law no. 196/2003 and EU GDPR 679/2016, respectively). The authorization (ref: 185046, 10/04/2019) for the execution of the research was given to us by the head teacher manager of the Comprehensive Scholastic Institute “E. Fermi” of Alba Adriatica (TE) who, according to Italian law, is primarily responsible for guaranteeing the ethical rights of children attending school in concomitance with the authorization of the experimental protocol from our university department (27/07/2019).

### 2.9. Statistical Analyses

We performed different statistical analyses. Descriptives and frequencies for demographic characteristics of the children and parents sample, of children's BMI and eating and life habits, and of parents' lifestyle and children's feeding styles. We reported descriptives and consistency measures (Cronbach's alpha) for children's and parents' assessments on psychological tests, and bivariate correlations were estimated between mothers' or fathers' psychological measures. A multigroup confirmatory factor analysis (MG-CFA) with a multiple indicator multiple cause model (MIMIC) was computed for testing measurement invariance between mother and father. We tested the measurement invariance using two CFA models, one in which the criterion is a factor representing children's mental state, measured by INT-CBCL, EXT-CBCL, and the OTHER, which are the components of the global score of the CBCL scale, and the other in which the criterion is a factor representing the parental stress measured by PD, PCDI, and DC, which are the components of the global score of the PSI scale. The predictors for the MIMIC models were the CEBQ subscales, which showed significant correlations with the total score of CBCL and PSI, both for mothers and fathers.

## 3. Results

Table 2 shows the frequencies and percentages of the values relative to the anthropometric data of the children, their lifestyle and food attitude, and the quality of their relationship with their parents. It also reports the frequency and percentages of lifestyle (sports, diet, and overweight) and feeding style of parents.

From the data in Table 2, it emerged that 78% of children played sports, 58% declared that eating is a pleasant experience, and 82% did not feel particularly overweight compared to their peers. On the basis of these declarations, the lifestyle of the 125 children can be considered healthy. This is also evidenced by the fact that only 13% of children had BMI values reporting obesity (only 5% of children were underweight). Regarding the quality of their relationship with their parents, most children reported that parents occasionally or often play with them (66% of mothers and 74% of fathers) and that parents were satisfied with them (69% of mothers and 68% of fathers were often satisfied with their children). Therefore, the relationship between parents and children showed a satisfactory quality. In relation to parents' lifestyle, 65% of them said they practiced sports at least occasionally, 75% avoided overeating, 52% followed a diet, and 93% had a normal weight or were moderately overweight. Therefore, parents also showed a substantially healthy lifestyle. Regarding parents' feeding habits, 27% of them often tried to convince their children to finish lunch or dinner, 82% cooked the dishes preferred by their children, and 62% declared that they let their children decide the food for lunch or dinner. Our data showed that parents' feeding habits were sufficiently healthy. There were no substantial differences between mothers and fathers in their lifestyle and feeding habits.

Table 3 shows the descriptive quantitative data of children (BMI score, Gen Anx, and Sep Anx) and of CEBQ, PSI-SF, and CBCL subscales, and total scores divided for mother and father. Cronbach's alpha and bivariate correlations with MC scores are reported to analyze the reliability of data.

Table 3 reports that the skewness and kurtosis values of the subscales of the various questionnaires, both for children and for parents, are included in the range of values −2 and +2. This means that data distributions of subjective responses are sufficiently normal [31]. Regarding reliability, most of Cronbach's alpha values are good (>0.79) or sufficient (>0.60). Only for some scales, the reliability values are lower than 0.60, especially in some subscales of the CBCL [32]. It is necessary to say that the children in our sample do not present clinical, mental, or behavioral disorders (less than 7% of children show severe symptoms). Since the CBCL is a questionnaire designed to detect the presence of clinical symptoms, its scores have greater consistency in the presence of severe clinical subjects [33, 34]. Regarding the correlation with the MC scale, both the PSI-SF and CBCL subscales show significant correlations, although all of them were below 0.50, indicating a weak correlation [35]. Thus, there is a weak effect of social desirability on parents' assessments of children's mental health and their stress. Such correlations are lower in fathers.

Table 4 shows the bivariate correlations between CEBQ, PSI-SF, and CBCL subscales for mothers (upper triangle) and fathers (lower triangle). In addition, the correlations between pairs of parents (number of pairs = 61) of various quantitative measures are also reported to analyze whether or not there is an agreement in parents' assessment of their children. The table also reports the correlations between BMI and children's responses to the anxiety test (Gen Anx, Sep Anx, and Tot Anx).

Table 4 shows the existence of significant intracorrelations between the PSI-SF and CBCL subscales. There is a strong convergence between the subscales that measure children's mental health and parental stress. There is also a strong intercorrelation between the CBCL subscale and the PSI-SF subscale. Other studies also showed that parents suffered from high levels of stress when their children had behavioral or psychological disorders [34]. With regard to the subscales of the CEBQ, there are no high intracorrelations between the subscales. The intercorrelations with the subscales of other questionnaires are significant for the CBCL and the PSI-SF subscales. Considering only the global score of the CBCL and PSI-SF, only the FR, EOE, DD, EUE, and FF subscales show significant correlations. Therefore, among the different eating habits measured by the CEBQ, the positive or negative reaction to food (FR or FF), the tendency to eat in relation to the emotional state (EOE or EUE), and the tendency to feel thirsty (DD) are the ones that are highly associated with children's mental health and parental stress.

Regarding the correlations between parental stress, children's mental health, and children's anxiety or BMI, no significant correlations emerged. It should be noted that only for mothers there are some significant correlations between some subscales of the CEBQ (FR, EOE, EF, SR, and SE) and the subscales of the PSI-SF with the BMI of the children.

Table 4 also shows the correlations between mothers' and fathers' assessments regarding the CEBQ, PSI-SF, and CBCL subscales. Almost all PSI-SF and CBCL subscales showed significant, although not very high, correlations (the highest correlation is 0.60 for INT-CBCL) both for mothers and fathers. The CEBQ subscales with significant correlations between mothers and fathers are DD, SE, EUE, and FF. Therefore, there is no strong convergence between mothers' and fathers' assessments of their children's eating habits.

There are strong intracorrelations between children's assessments of anxiety (Gen Anx, Sep Anx, and Tot Anx), but none of these are significantly correlated with BMI.

**Figure 1:**
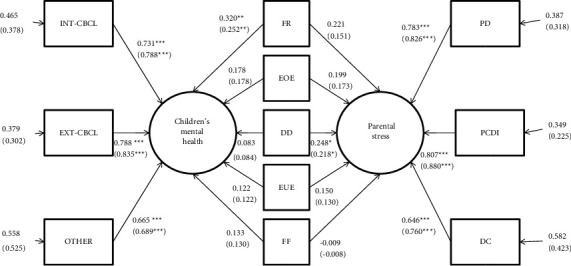
Path diagram of the MIMIC models for the analysis of the relationships between the evaluation of eating habits and the evaluations of the mental health of children and parental stress. Standardized parameters of loadings, regression coefficients, and residuals are reported for mothers (fathers). ^*∗∗∗*^significant at 0.001 level, ^*∗∗*^significant at 0.01 level, and ^*∗*^significant at 0.05 level. Children's eating behavior subscales: FR: food responsiveness, EOE: emotional overeating, EF: enjoyment of food, DD: desire to drink, EUE: emotional undereating, FF: food fussiness; parenting stress index subscales: PD: parental stress, PDCI: parent-child dysfunctional interaction, DC: difficult child; and child behavior check list subscales: OTHER: other problems, INT-CBCL: internalizing CBCL symptoms, and EXT-CBCL: externalizing CBCL symptoms.

Bivariate correlations showed that FR, EOE, DD, EUE, and FF are the eating habits that have a significant correlation with children's mental health and parental stress. All these subscales are indicators of unhealthy eating habits. Therefore, these subscales are used as predictors of the global level of children's mental health and parental stress. Global mental health is measured by the indices of internalizing (INT-CBCL) and externalized (EXT-CBCL) symptoms and of other symptoms (OTHER) that make up the global CBCL score, while the global parental stress is measured by the three subscales of the PSI-SF (PD, PCDI, and DC).  Figure 1 shows the MIMIC models tested in our analyses.

The measurement invariance of the MIMIC models was tested using the chi-square difference between the model with free parameters and fixed parameters. The first model (M_free_) is the comparison model with free parameters for both groups. The second model tested loading invariance between groups (M_load_). The third model tested loading and intercepts invariance between groups (M_load_ _+_ _int_). The fourth model tested loadings, intercepts, and regression coefficient invariance between groups (M_load_ _+_ _int_ _+_ _reg_). If the chi-square difference (Δ*χ*2) between nested models is not significant (*p* > 0.05), then the measurement invariance is confirmed [36]. We also calculated the comparative fit index (CFI) of each model, and we used the difference of CFI (ΔCFI) to test measurement invariance. According to Chen [37], a difference of less than 0.01 (in absolute values) indicates measurement invariance.

Table 5 shows the results of the measurement invariance analysis between the MIMIC models. All Δ*χ*2 are not significant, and all the ΔCFI were <|0.01|, confirming the measurement invariance of MIMIC models. Therefore, there is an agreement between mothers and fathers in judging the mental health problems of their children and their own level of parental stress.  Figure 1 shows the standardized parameters (loading and regression coefficients), both for mothers and fathers, and the relative significance (*z*-test) of each parameter.

Among eating habits, only FR resulted to be significantly predictive of mental health (CBCL), and only DD resulted to be significantly predictive of parental stress (PSI). FR is the principal eating behavior connected to presence of behavioral or psychological problems in children, while DD is the eating behavior with the strongest connection with parental stress.

## 4. Discussion

Our study has shown that parents' assessments of their children's eating disorders are consistent and reliable. Their assessments of children's mental health and parental stress are also reliable.

Hypothesis H1a was partially confirmed. Children's eating habits are not affected by children's BMI, except for mothers. Our study shows a higher sensitivity of mothers toward the physical fitness of their children, indicated by the significant correlations between children's eating habits and parental stress on the BMI index, while fathers seem to neglect this connection. The greater attention of mothers to the physical fitness of their children has also been highlighted by other studies [14, 16]. Probably, it is due to mothers' and fathers' different social and cultural attitudes regarding the physical aspect of their children [10, 16, 17]. This seems the most probable explanation because, in our sample, both mothers and fathers apply the same way of feeding their children unlike the evidences of other studies [14–16, 22]. Despite these differences, however, mothers and fathers both prove to be good observers of their child's eating behavior.

Hypothesis H1b was not confirmed. Children's anxiety (general and separation anxiety) is not correlated to their eating habits.

Hypothesis H2a was confirmed only for FR, and hypothesis H2b was confirmed only for DD. Eating habits have the same predictive value on children's mental health and parental stress, both for mothers and fathers. In particular, children show better mental health if they like and enjoy eating. The high desire to drink in children is significantly related to a higher level of stress and discomfort in parents. Therefore, according to parents' assessment, children's positive attitude toward food is associated with good and well-adapted behavior, while excessive desire in eating or drinking can generate anxiety and stress in parents. There are no differences between mothers and fathers in their cognitive or emotive reactions to their children's eating habits. Substantially, our data confirm the results of previous studies that found a positive association between children's mental health and healthy eating habits [5–7]. Our data also confirm the negative impact of children's unhealthy eating habits on parents' psychological well-being [10]. The fact that only some dimensions of children's eating habits are related to children's mental health and parental stress can be explained by the low percentage of children with serious eating problems in our sample.

A limit to our research is due to the fact that the subjects are all part of a restricted geographical complex, given that they were selected within a specific scholastic complex. However, it is quite difficult to recruit volunteers for research purposes without the help of an institution operating on the territory such as a school in this case. Another point is that this study should be extended to children who really suffer from severe eating disorders to see how, in this case, parents' assessment of their children's eating habits and behavior change in relation to their problems. It must be said, however, that from a preventive perspective, it is necessary to involve also families with healthy eating habits as control groups in programs for the identification of possible forms of unhealthy eating behavior to prevent the appearance of more serious problems, which then require medical and/or psychological intervention or rehabilitation [4, 7, 10].

## 5. Conclusion

The results of our study show that parents can play an important role in assessing their children's eating habits. Parents can assist pediatricians, psychiatrists, and clinical psychologists of the developmental age in diagnosing the presence of eating disorders, which can then affect both the behavior and mental state of children and the psychological well-being of parents. The risk of obesity is a real risk in today's society [38] [2]. In particular, in Italy, 32.4% of male children and 30.9% of female children are overweight or obese [39]. A quick and effective intervention by pediatricians, psychiatrists, and psychologists is essential to avoid the appearance and persistence of unhealthy eating habits [4]. Parents can play an important role in preventing obesity, both because they give primal care to their children's health and because they are constantly in contact with them. Parents, unlike other caregivers or professional figures, have a greater ability to grasp and detect the presence of an eating disorder in their children [10, 16]. In addition, variations in life habits due to exceptional events, such as the COVID-19 pandemic, that forced people to live in isolation, make every single family essential to ensure correct eating habits in children [40].

Other studies are needed to define better intervention programs to prevent childhood obesity, but our study highlights that in these programs, the involvement of parents in the observation and assessment of children's eating habits is necessary and affordable.

## Figures and Tables

**Table 1:** Demographic characteristics of parents (*n* = 161) who participated in the study.

		Frequency	Percentage
Civil status	Married	149	93
	Separated or divorced	12	7
Education level	Primary school	5	3
	Secondary school	29	18
	High school	82	51
	University degree	35	22
	Missing	10	6
Occupation	Not qualified job (e.g., porter, day laborer, etc.)	2	1
	Seasonal worker	7	4
	Farmer	1	1
	Home worker	27	17
	Retailer or trade worker	11	7
	Clerk or administrative worker	23	14
	Teacher or professor	5	3
	Dependent worker	42	26
	Autonomous worker or professional	31	19
	Manager or head office	6	4
	Missing	6	4
Annual income	<Є10,000	30	19
	Є10,000–Є25,000	66	41
	Є25,000–Є40,000	21	13
	Є40,000–Є55,000	4	2
	>Є55,000	6	4
	Missing	34	21

**Table 2:** Frequencies and percentages of anthropometric data lifestyle, food attitude, and parental relations of the children (*n* = 125) and frequencies and percentages of lifestyle (sports, diet and overweight) and feeding style of parents (*n* = 161). Children's body mass index (BMI) categories are also reported.

*Children's lifestyle*				
“Do you play sports?”	Yes	98 (78%)		
	No	27 (22%)		
“Do you like eating?”	Yes	72 (58%)		
	No	53 (42%)		
“Do you feel fatter than other children?”	Yes	22 (18%)		
	No	103 (82%)		

*Children-parents relation*				
“Does your mother show that she is satisfied with you?”	Never	5 (4%)		
	Occasionally	34 (27%)		
	Often	86 (69%)		
“Does your mother play with you?	Never	41 (33%)		
	Occasionally	63 (50%)		
	Often	20 (16%)		
	Missing	1 (1%)		
“Does your father show that he is satisfied with you?”	Never	6 (5%)		
	Occasionally	34 (27%)		
	Often	85 (68%)		
“Does your father play with you?	Never	32 (26%)		
	Occasionally	61 (49%)		
	Often	32 (26%)		
BMI categories	Underweight (<18)	6 (5%)		
	Normal (18–25)	86 (69%)		
	Overweight (25–30)	17 (13%)		
	Obese (>30)	16 (13%)		

*Parents' lifestyle*		*All*	*Mothers*	*Fathers*
“Do you play sports?”	Never	57 (35%)	35 (38%)	22 (32%)
	Occasionally	79 (49%)	43 (47%)	36 (52%)
	Often	25 (16%)	14 (15%)	11 (16%)
“Do you eat too much?”	Yes	40 (25%)	15 (16%)	25 (36%)
	No	121 (75%)	77 (84%)	44 (64%)
“Do you follow a diet?”	Yes	84 (52%)	54 (59%)	30 (43%)
	No	77 (4%)	38 (41%)	39 (57%)
“Are you overweight?”	No	73 (45%)	46 (50%)	27 (39%)
	Moderately	77 (48%)	42 (46%)	35 (51%)
	Strongly	11 (7%)	4 (4%)	7 (10%)

*Parents' feeding style*				
“Do you try to get your child to finish lunch when s/he is not eating?”	Never	34 (21%)	17 (18%)	17 (25%)
	Occasionally	83 (52%)	49 (53%)	34 (49%)
	Often	44 (27%)	26 (28%)	18 (26%)
“Do you cook the dishes your child likes more?”	Never	29 (18%)	15 (16%)	14 (20%)
	Occasionally	108 (67%)	62 (67%)	46 (67%)
	Often	24 (15%)	15 (16%)	9 (13%)
“Is your child deciding what to eat?”	Never	52 (32%)	30 (33%)	22 (32%)
	Occasionally	100 (62%)	56 (61%)	44 (64%)
	Often	9 (6%)	6 (7%)	3 (4%)

**Table 3:** Descriptives of quantitative data for children (BMI score, Gen Anx, Sep Anx, and Tot Anx) and of CEBQ, PSI-SF, CBCL subscales, and total scores divided for mothers and fathers. Cronbach's alphas and bivariate correlations with MC scores are also reported. Fathers' values are reported in parentheses.

	Mean	SD	Skewness	Kurtosis	Cronbach's alphas	Correlations with MC
*Children*						
BMI scores	18.61	3.56	0.89	0.21		
Gen Anx	3.94	1.89	0.11	−0.46	0.47	
Sep Anx	4.44	2.17	0.06	−0.20	0.53	
Tot Anx	8.31	3.29	0.19	−0.14	0.60	

*Parents CEBQ subscales*						
FR	10.02 (6.81)	7.21 (4.49)	0.18 (0.27)	−1.67 (−1.49)	0.69 (0.68)	−0.36^*∗∗*^(−0.04)
EOE	7.96 (7.88)	3.82 (4.41)	−0.45 (−0.22)	−1.12 (−1.49)	0.75 (0.74)	−0.18 (−0.24^*∗*^)
EF	7.14 (5.43)	5.08 (3.98)	0.49 (0.77)	−1.29 (−0.84)	0.58 (0.56)	−0.20 (0.12)
DD	7.15 (6.48)	2.58 (2.23)	−0.70 (−0.50)	0.24 (−0.18)	0.59 (0.61)	−0.28^*∗*^(−0.08)
SR	6.52 (5.87)	3.71 (2.75)	0.70 (0.39)	−0.16 (−0.45)	0.46 (0.52)	0.02 (0.14)
SE	8.07 (6.09)	5.65 (4.42)	0.16 (0.43)	−1.64 (−1.44)	0.62 (0.68)	−0.01 (0.12)
EUE	7.45 (7.52)	2.62 (2.73)	−0.79 (−0.94)	0.10 (0.20)	0.54 (0.29)	−0.22^*∗*^(−0.15)
FF	10.73 (10.55)	5.19 (4.79)	0.44 (0.50)	−0.47 (−0.38)	0.78 (0.80)	−0.13 (−0.24)

*PSI-SF subscales and total score*						
PD	14.41 (11.07)	8.97 (7.42)	0.51 (0.65)	−0.71 (−0.66)	0.86 (0.85)	−0.42^*∗∗∗*^(−0.27^*∗*^)
PDCI	9.37 (7.67)	7.12 (5.67)	0.71 (0.84)	−0.69 (−0.43)	0.88 (0.84)	−0.30^*∗∗*^ (−0.19)
DC	12.67 (9.84)	7.88 (6.40)	0.34 (0.35)	−1.00 (−0.95)	0.86 (0.85)	−0.35^*∗∗*^(−0.19)
Tot PSI	28.10 (21.28)	14.64 (11.20)	0.11 (0.02)	−1.00 (−1.11)	0.94 (0.93)	−0.43^*∗∗∗*^(−0.25^*∗*^)

*CBCL 6/18 subscales and total score*						
INT	3.63 (2.68)	2.98 (2.00)	1.37 (1.41)	0.91 (1.54)	0.58 (0.66)	−0.30^*∗∗*^(−0.08)
SOM	3.25 (2.36)	2.85 (1.98)	1.26 (1.52)	0.43 (1.70)	0.69 (0.62)	−0.10 (0.01)
ANX	8.54 (6.07)	5.09 (3.90)	−0.18 (0.24)	−1.23 (−0.94)	0.74 (0.76)	−0.26^*∗*^(−0.08)
SOC	6.68 (5.23)	4.82 (3.69)	0.27 (0.32)	−1.20 (−1.13)	0.81 (0.78)	−0.36^*∗∗*^(−0.19)
IDE	2.49 (1.99)	2.22 (1.68)	1.61 (2.13)	1.25 (4.04)	0.45 (0.53)	−0.25^*∗*^(−0.20)
ATT	8.21 (7.64)	4.72 (3.98)	−0.15 (−0.15)	−1.30 (−0.86)	0.64 (0.69)	−0.39^*∗∗∗*^(−0.17)
DEV	2.28 (2.09)	1.55 (1.44)	1.16 (1.82)	0.34 (3.04)	0.29 (0.14)	−0.31^*∗∗*^(−0.25^*∗*^)
AGG	11.48 (9.10)	6.21 (4.44)	−0.31 (−0.41)	−1.28 (−0.80)	0.78 (0.74)	−0.34^*∗∗*^(−0.05)
OTHER	3.16 (3.39)	3.16 (2.49)	0.68 (1.03)	−0.50 (0.29)	0.30 (0.45)	−0.38^*∗∗∗*^(−0.13)
INT-CBCL	11.29 (12.68)	6.70 (7.06)	−0.24 (−0.55)	−1.43 (−1.17)	0.80 (0.83)	−0.26^*∗*^(−0.06)
EXT-CBCL	11.62 (10.80)	6.02 (5.19)	−0.30 (−0.52)	−1.18 (−0.85)	0.80 (0.75)	−0.37^*∗∗*^(−0.11)
Tot CBCL	19.02 (16.28)	11.87 (10.7)	0.15 (0.20)	−1.40 (−1.45)	0.88 (0.89)	−0.38^*∗∗*^(−0.12)
MC	34.45 (34.06)	4.97 (5.38)	−0.13 (0.16)	−0.57 (−0.89)	0.62 (0.51)	

*Note*: BMI: body mass index, Gen Anx: general anxiety level (in children), Sep Anx: separation anxiety (in children), Tot Anx: global score of anxiety (in children), CEBQ: children's eating behavior questionnaire, FR: food responsiveness. EOE: emotional overeating, EF: enjoyment of food, DD: desire to drink, SR: satiety responsiveness, SE: slowness in eating, EUE: emotional undereating, FF: food fussiness, PSI-SF: parenting stress index-short form, PD: parental stress, PDCI: parent-child dysfunctional interaction, DC: difficult child, Tot PSI: global score of the PSI-SF scale, CBCL: child behavior check list, INT: introversion, SOM: psychosomatic disorders, ANX: anxiety and depression, SOC: socialization, IDE: ideation, ATT: attention, DEV: deviant behavior, AGG: aggression and hyperactivity, OTHER: other problems, INT-CBCL: internalizing CBCL symptoms, EXT-CBCL: externalizing CBCL symptoms, Tot CBCL: global CBCL score, and MC: Marlowe–Crowne scale for social desirability.

**Table 4:** Bivariate correlations between CEBQ, PSI-SF, and CBCL subscales for mothers (upper triangle) and fathers (lower triangle) and between parents' scales and children's scales (Gen Anx, Sep Anx, and Tot Anx) and BMI. In addition, the correlations between the pairs of parents (number of pairs = 61) are reported (M-F correlations).

		1	2	3	4	5	6	7	8	9	10	11	12	13	14	15	16	17	18	19	20	21	22	23	24	25	26	27	28
1	FR		0.51^*∗∗∗*^	0.50^*∗∗∗*^	0.37^*∗∗∗*^	−0.28^*∗∗*^	−0.24^*∗*^	0.15	−0.14	0.43^*∗∗∗*^	0.34^*∗∗*^	0.36^*∗∗*^	0.45^*∗∗∗*^	0.16	0.20	0.18	0.32^*∗∗*^	0.18	0.22^*∗*^	0.32^*∗∗*^	0.43^*∗∗∗*^	0.36^*∗∗*^	0.22	0.45^*∗∗∗*^	0.40^*∗∗∗*^	−0.01	0.13	0.09	0.49^*∗∗∗*^
2	EOE	0.54^*∗∗∗*^		0.28^*∗∗*^	0.27^*∗∗*^	−0.13	−0.25^*∗*^	0.46^*∗∗∗*^	−0.10	0.39^*∗∗∗*^	0.44^*∗∗∗*^	0.31^*∗∗*^	0.45^*∗∗∗*^	0.15	0.23^*∗*^	0.33^*∗∗*^	0.29^*∗∗*^	0.22^*∗*^	0.29^*∗∗*^	0.39^*∗∗∗*^	0.33^*∗∗*^	0.34^*∗∗*^	0.33^*∗∗*^	0.37^*∗∗*^	0.42^*∗∗∗*^	0.16	0.24^*∗*^	0.26^*∗*^	0.37^*∗∗∗*^
3	EF	0.29^*∗*^	0.15		0.09	−0.42^*∗∗∗*^	−0.35^*∗∗*^	0.23^*∗*^	−0.40^*∗∗∗*^	0.20	0.03	0.07	0.12	−0.05	0.00	0.03	0.05	0.00	0.07	0.16	0.12	0.10	0.00	0.14	0.11	−0.04	0.11	0.05	0.27^*∗*^
4	DD	0.46^*∗∗∗*^	0.50^*∗∗∗*^	0.19		0.16	0.27^*∗*^	0.11	0.12	0.33^*∗∗*^	0.35^*∗∗*^	0.33^*∗∗*^	0.4^*∗∗∗*^	0.21	0.05	0.27^*∗*^	0.27^*∗*^	0.17	0.24^*∗*^	0.09	0.26^*∗*^	0.17	0.27^*∗*^	0.25^*∗*^	0.30^*∗∗*^	0.06	0.01	0.05	0.20
5	SR	0.06	0.03	−0.18	0.08		0.42^*∗∗∗*^	−0.01	0.26^*∗*^	−0.15	−0.06	0.14	−0.03	−0.05	0.05	0.10	0.19	0.14	0.14	−0.01	0.05	−0.02	0.04	0.05	0.02	−0.04	0.05	0.02	−0.23^*∗*^
6	SE	−0.17	−0.2	−0.06	−0.20	0.20		−0.07	0.30^*∗∗*^	−0.18	0.04	−0.09	−0.10	0.16	−0.03	0.11	0.10	−0.01	−0.09	−0.21	−0.17	−0.07	0.13	−0.2	−0.05	−0.09	0.12	0.03	−0.28^*∗∗*^
7	EUE	0.23	0.39^*∗∗*^	0.02	0.16	0.24^*∗*^	0.00		0.08	0.22^*∗*^	0.28^*∗∗*^	0.15	0.25^*∗*^	0.18	0.27^*∗*^	0.36^*∗∗*^	0.21	0.13	0.09	0.11	0.11	0.12	0.35^*∗∗*^	0.12	0.26^*∗*^	0.03	0.06	0.05	0.12
8	FF	0.19	0.18	−0.26^*∗*^	0.16	0.1	0.15	0.12		−0.13	−0.07	0.02	−0.07	0.09	0.07	0.11	0.04	0.06	−0.04	−0.09	0.00	0.12	0.10	−0.01	0.06	0.06	0.07	0.07	−0.17
9	PD	0.24^*∗*^	0.32^*∗∗*^	−0.13	0.32^*∗∗*^	0.13	−0.05	0.23	0.26^*∗*^		0.69^*∗∗∗*^	0.52^*∗∗∗*^	0.87^*∗∗∗*^	0.42^*∗∗∗*^	0.22^*∗*^	0.42^*∗∗∗*^	0.4^*∗∗∗*^	0.36^*∗∗*^	0.38^*∗∗∗*^	0.36^*∗∗*^	0.44^*∗∗∗*^	0.46^*∗∗∗*^	0.45^*∗∗∗*^	0.46^*∗∗∗*^	0.55^*∗∗∗*^	0.04	0.01	0.01	0.33^*∗∗*^
10	PCDI	0.28^*∗*^	0.32^*∗∗*^	−0.13	0.22	0.18	0.09	0.24^*∗*^	0.17	0.68^*∗∗∗*^		0.55^*∗∗∗*^	0.86^*∗∗∗*^	0.48^*∗∗∗*^	0.25^*∗*^	0.43^*∗∗∗*^	0.39^*∗∗∗*^	0.37^*∗∗*^	0.37^*∗∗*^	0.33^*∗∗*^	0.28^*∗∗*^	0.42^*∗∗∗*^	0.51^*∗∗∗*^	0.32^*∗∗*^	0.51^*∗∗∗*^	0.06	0.02	0.04	0.21^*∗*^
11	DC	0.25^*∗*^	0.23	−0.18	0.09	0.14	0.04	0.17	0.19	0.56^*∗∗∗*^	0.64^*∗∗∗*^		0.82^*∗∗∗*^	0.22	0.36^*∗∗*^	0.4^*∗∗∗*^	0.55^*∗∗∗*^	0.29^*∗∗*^	0.47^*∗∗∗*^	0.45^*∗∗∗*^	0.68^*∗∗∗*^	0.44^*∗∗∗*^	0.41^*∗∗∗*^	0.70^*∗∗∗*^	0.62^*∗∗∗*^	0.06	−0.05	−0.01	0.23^*∗*^
12	Tot PSI	0.30^*∗*^	0.33^*∗∗*^	−0.17	0.24^*∗*^	0.17	0.03	0.24^*∗*^	0.24^*∗*^	0.88^*∗∗∗*^	0.88^*∗∗∗*^	0.85^*∗∗∗*^		0.44^*∗∗∗*^	0.33^*∗∗*^	0.49^*∗∗∗*^	0.53^*∗∗∗*^	0.4^*∗∗∗*^	0.49^*∗∗∗*^	0.45^*∗∗∗*^	0.56^*∗∗∗*^	0.52^*∗∗∗*^	0.54^*∗∗∗*^	0.60^*∗∗∗*^	0.66^*∗∗∗*^	0.06	−0.01	0.02	0.31^*∗∗*^
13	INT	0.17	0.14	−0.1	0.18	−0.10	0.06	0.15	0.28^*∗*^	0.46^*∗∗∗*^	0.56^*∗∗∗*^	0.45^*∗∗∗*^	0.55^*∗∗∗*^		0.22^*∗*^	0.49^*∗∗∗*^	0.42^*∗∗∗*^	0.33^*∗∗*^	0.44^*∗∗∗*^	0.18	0.3^*∗∗*^	0.3^*∗∗*^	0.67^*∗∗∗*^	0.30^*∗∗*^	0.55^*∗∗∗*^	0.14	0.1	0.13	0.06
14	SOM	0.31^*∗*^	0.17	−0.14	0.07	0.17	−0.06	0.10	0.12	0.18	0.30^*∗*^	0.26^*∗*^	0.28^*∗*^	0.13		0.61^*∗∗∗*^	0.6^*∗∗∗*^	0.45^*∗∗∗*^	0.49^*∗∗∗*^	0.36^*∗∗*^	0.45^*∗∗∗*^	0.38^*∗∗∗*^	0.73^*∗∗∗*^	0.47^*∗∗∗*^	0.66^*∗∗∗*^	0.14	0.23^*∗*^	0.23^*∗*^	−0.10
15	ANX	0.30^*∗*^	0.32^*∗∗*^	−0.04	0.24	0.10	0.04	0.28^*∗*^	0.19	0.51^*∗∗∗*^	0.64^*∗∗∗*^	0.56^*∗∗∗*^	0.65^*∗∗∗*^	0.56^*∗∗∗*^	0.45^*∗∗∗*^		0.76^*∗∗∗*^	0.57^*∗∗∗*^	0.62^*∗∗∗*^	0.37^*∗∗*^	0.56^*∗∗∗*^	0.43^*∗∗∗*^	0.94^*∗∗∗*^	0.58^*∗∗∗*^	0.84^*∗∗∗*^	0.12	0.23^*∗*^	0.20	0.00
16	SOC	0.35^*∗∗*^	0.36^*∗∗*^	0.04	0.30^*∗*^	0.05	−0.03	0.18	0.15	0.53^*∗∗∗*^	0.58^*∗∗∗*^	0.56^*∗∗∗*^	0.64^*∗∗∗*^	0.45^*∗∗∗*^	0.5^*∗∗∗*^	0.82^*∗∗∗*^		0.48^*∗∗∗*^	0.69^*∗∗∗*^	0.46^*∗∗∗*^	0.6^*∗∗∗*^	0.46^*∗∗∗*^	0.76^*∗∗∗*^	0.63^*∗∗∗*^	0.77^*∗∗∗*^	0.14	0.24^*∗*^	0.23^*∗*^	0.18
17	IDE	0.12	0.27^*∗*^	−0.04	0.29^*∗*^	0.04	−0.06	0.20	0.16	0.58^*∗∗∗*^	0.42^*∗∗∗*^	0.26^*∗*^	0.49^*∗∗∗*^	0.25^*∗*^	0.25^*∗*^	0.43^*∗∗∗*^	0.48^*∗∗∗*^		0.49^*∗∗∗*^	0.37^*∗∗∗*^	0.37^*∗∗*^	0.39^*∗∗∗*^	0.58^*∗∗∗*^	0.41^*∗∗∗*^	0.56^*∗∗∗*^	0.01	−0.04	−0.01	0.13
18	ATT	0.15	0.28^*∗*^	−0.18	0.24^*∗*^	0.07	−0.05	0.22	0.16	0.59^*∗∗∗*^	0.56^*∗∗∗*^	0.53^*∗∗∗*^	0.65^*∗∗∗*^	0.42^*∗∗∗*^	0.43^*∗∗∗*^	0.67^*∗∗∗*^	0.71^*∗∗∗*^	0.36^*∗∗*^		0.4^*∗∗∗*^	0.57^*∗∗∗*^	0.41^*∗∗∗*^	0.66^*∗∗∗*^	0.59^*∗∗∗*^	0.70^*∗∗∗*^	0.22	0.19	0.22^*∗*^	0.12
19	DEV	0.20	0.29^*∗*^	0.04	0.30^*∗*^	−0.08	−0.19	0.09	0.04	0.54^*∗∗∗*^	0.43^*∗∗∗*^	0.46^*∗∗∗*^	0.56^*∗∗∗*^	0.17	0.30^*∗*^	0.38^*∗∗*^	0.52^*∗∗∗*^	0.58^*∗∗∗*^	0.43^*∗∗∗*^		0.45^*∗∗∗*^	0.42^*∗∗∗*^	0.38^*∗∗*^	0.61^*∗∗∗*^	0.58^*∗∗∗*^	0.04	0.07	0.07	0.11
20	AGG	0.28^*∗*^	0.28^*∗*^	−0.14	0.26^*∗*^	−0.01	−0.01	0.22	0.25^*∗*^	0.42^*∗∗∗*^	0.47^*∗∗∗*^	0.60^*∗∗∗*^	0.57^*∗∗∗*^	0.4^*∗∗*^	0.40^*∗∗*^	0.64^*∗∗∗*^	0.67^*∗∗∗*^	0.41^*∗∗*^	0.64^*∗∗∗*^	0.36^*∗∗*^		0.54^*∗∗∗*^	0.57^*∗∗∗*^	0.98^*∗∗∗*^	0.86^*∗∗∗*^	0.06	0.11	0.1	0.21
21	OTHER	0.28^*∗*^	0.29^*∗*^	−0.02	0.23	0.06	0.13	0.21	0.26^*∗*^	0.47^*∗∗∗*^	0.58^*∗∗∗*^	0.36^*∗∗*^	0.54^*∗∗∗*^	0.35^*∗∗*^	0.30^*∗*^	0.54^*∗∗∗*^	0.50^*∗∗∗*^	0.53^*∗∗∗*^	0.57^*∗∗∗*^	0.37^*∗∗*^	0.49^*∗∗∗*^		0.46^*∗∗∗*^	0.56^*∗∗∗*^	0.71^*∗∗∗*^	0.13	0.11	0.16	0.24^*∗*^
22	INT-CBCL	0.32^*∗∗*^	0.30^*∗*^	−0.10	0.23	0.10	0.03	0.24	0.24^*∗*^	0.52^*∗∗∗*^	0.67^*∗∗∗*^	0.57^*∗∗∗*^	0.66^*∗∗∗*^	0.67^*∗∗∗*^	0.65^*∗∗∗*^	0.94^*∗∗∗*^	0.80^*∗∗∗*^	0.44^*∗∗∗*^	0.68^*∗∗∗*^	0.40^*∗∗*^	0.65^*∗∗∗*^	0.55^*∗∗∗*^		0.58^*∗∗∗*^	0.88^*∗∗∗*^	0.16	0.25^*∗*^	0.24^*∗*^	−0.03
23	EXT-CBCL	0.29^*∗*^	0.32^*∗∗*^	−0.11	0.30^*∗*^	−0.03	−0.06	0.22	0.23	0.51^*∗∗∗*^	0.53^*∗∗∗*^	0.64^*∗∗∗*^	0.65^*∗∗∗*^	0.4^*∗∗*^	0.42^*∗∗∗*^	0.66^*∗∗∗*^	0.72^*∗∗∗*^	0.50^*∗∗∗*^	0.67^*∗∗∗*^	0.57^*∗∗∗*^	0.97^*∗∗∗*^	0.53^*∗∗∗*^	0.68^*∗∗∗*^		0.88^*∗∗∗*^	0.06	0.11	0.10	0.21
24	Tot CBCL	0.36^*∗∗*^	0.34^*∗∗*^	−0.1	0.28^*∗*^	0.03	0.04	0.28^*∗*^	0.29^*∗*^	0.58^*∗∗∗*^	0.68^*∗∗∗*^	0.65^*∗∗∗*^	0.73^*∗∗∗*^	0.59^*∗∗∗*^	0.58^*∗∗∗*^	0.88^*∗∗∗*^	0.82^*∗∗∗*^	0.55^*∗∗∗*^	0.75^*∗∗∗*^	0.52^*∗∗∗*^	0.85^*∗∗∗*^	0.70^*∗∗∗*^	0.92^*∗∗∗*^	0.88^*∗∗∗*^		0.17	0.19	0.21	0.11
25	Gen Anx	0.18	0.18	0.17	0.15	−0.06	−0.12	0.03	0.18	0.09	0.12	0.08	0.11	0.22	0.15	0.10	0.06	0.07	0.06	0.15	0.15	0.13	0.17	0.17	0.19		0.32^*∗∗∗*^	0.79^*∗∗∗*^	0.12
26	Sep Anx	0.23	0.28^*∗*^	0.17	0.04	0.11	0.04	0.20	0.14	−0.04	0.03	−0.07	−0.03	0.15	0.28^*∗*^	0.17	0.21	−0.05	0.12	0.02	0.05	0.04	0.24	0.05	0.17			0.84^*∗∗∗*^	−0.00
27	Tot Anx	0.28^*∗*^	0.29^*∗*^	0.23	0.14	0.04	−0.04	0.13	0.20	0.03	0.10	0.00	0.04	0.22	0.28^*∗*^	0.17	0.18	0.01	0.08	0.11	0.12	0.11	0.26^*∗*^	0.13	0.22				0.07
28	BMI	0.21	0.17	0.17	0.2	−0.06	−0.12	−0.09	0.05	0.24^*∗*^	0.14	0.05	0.17	0.09	−0.24^*∗*^	−0.07	0.00	0.12	0.01	0.16	0.01	0.10	−0.09	0.05	−0.01				
	M-F correlations	0.24	0.19	0.08	0.32^*∗*^	0.25	0.32^*∗*^	0.31^*∗*^	0.29^*∗*^	0.31^*∗*^	0.44^*∗∗∗*^	0.42^*∗∗∗*^	0.42^*∗∗∗*^	0.48^*∗∗∗*^	0.35^*∗∗*^	0.45^*∗∗∗*^	0.44^*∗∗∗*^	0.53^*∗∗∗*^	0.35^*∗∗*^	0.22	0.35^*∗∗*^	0.40^*∗∗*^	0.60^*∗∗∗*^	0.34^*∗∗*^	0.50^*∗∗∗*^				

*Note*: ^*∗∗∗*^Significant at 0.001 level, ^*∗∗*^significant at 0.01 level, and ^*∗*^significant at 0.05 level. BMI: body mass index, Gen Anx: general anxiety level (in children), Sep Anx: separation anxiety (in children), Tot Anx: global score of anxiety (in children), CEBQ: children's eating behavior questionnaire, FR: food responsiveness, EOE: emotional overeating, EF: enjoyment of food, DD: desire to drink, SR: satiety responsiveness, SE: slowness in eating, EUE: emotional undereating, FF: food fussiness, PSI-SF: parenting stress index-short form, PD: parental stress, PDCI: parent-child dysfunctional interaction, DC: difficult child, Tot PSI: global score of the PSI-SF scale, CBCL: child behavior check list, INT: introversion, SOM: psychosomatic disorders, ANX: anxiety and depression, SOC: socialization, IDE: ideation, ATT: attention, DEV: deviant behavior, AGG: aggression and hyperactivity, OTHER: other problems, INT-CBCL: internalizing CBCL symptoms, EXT-CBCL: externalizing CBCL symptoms, and Tot CBCL: global CBCL score.

**Table 5:** Multigroup invariance measurement of MIMIC models M_free_, M_load_, M_load_ _+_ _int_, and M_load_ _+_ _int_ _+_ _reg_, between mothers and fathers both for children's mental health (CBCL) and for parental stress (PSI).

	AIC	BIC	*χ* ^2^	df	CFI	Model comparisons	*χ* ^2^ difference	df difference	*P* (*χ*^2^ difference)	ΔCFI
*Children's mental health (CBCL) MIMIC model*										
M_free_	2,236.7	2,319.1	20.866	20	0.994					
M_load_	2,235.4	2,311.8	23.534	22	0.989	M_free_−M_load_	2.6684	2	0.26	−0.005
M_load + int_	2,233.0	2,303.6	25.163	24	0.992	M_load_−M_load+int_	1.6285	2	0.44	0.003
M_load + int + reg_	2,224.1	2,280.0	26.308	29	0.999	M_load+int_−M_load+int+reg_	1.1456	5	0.95	0.007

*Parental stress (PSI) MIMIC model*										
M_free_	2,743.3	2,825.7	18.266	20	0.999					
M_load_	2,739.9	2,816.4	18.903	22	0.999	M_free_−M_load_	0.6374	2	0.72	<0.0001
M_load + int_	2,737.1	2,807.7	20.036	24	0.999	M_load_−M_load + int_	1.1329	2	0.56	<0.0001
M_load + int + reg_	2,733.3	2,789.2	26.256	29	0.998	M_load + int_−M_load + int + reg_	6.2195	5	0.28	0.001

*Note*: MIMIC: multiple indicator multiple cause model, M_free_: MIMIC model with free parameters for each group, M_load_: MIMIC model with loadings invariance for each group, M_load+int_: MIMIC model with loadings and intercepts invariance for each group, M_load+int+reg_: MIMIC model with loadings, intercepts, and regression coefficients invariance for each group, AIC: Akaike's information criterion, BIC: Bayesian information criterion, CFI: comparative fit index, ΔCFI: difference in CFI values, CBCL: child behavior check list, and PSI: parenting stress index.

## Data Availability

The data sets used and/or analyzed during the current study are available from the corresponding author on reasonable request.
